# *Adenia sajoreciae* (Passifloraceae), a new species from Kenya and its phylogenetic placement

**DOI:** 10.3897/phytokeys.276.190059

**Published:** 2026-06-15

**Authors:** Emmanuel Kipkemoi, Fredrick Munyao Mutie, Xiang-Dong Qiu, Paul Muigai Kirika, John Kamau Muchuku, Elijah Nyakudya, Xue Yan, Sheng-Wei Wang, Neng Wei, Qing-Feng Wang

**Affiliations:** 1 State Key Laboratory of Plant Diversity and Specialty Crops, Wuhan Botanical Garden, Chinese Academy of Sciences, Wuhan 430074, Hubei, China Jomo Kenyatta University of Agriculture and Technology Nairobi Kenya; 2 University of Chinese Academy of Sciences, Beijing 100049, China Wuhan Botanical Garden, Chinese Academy of Sciences Wuhan China; 3 Sino-Africa Joint Research Centre (SAJOREC), Chinese Academy of Sciences, Wuhan 430074, Hubei, China National Museums of Kenya Nairobi Kenya; 4 School of Ecology and Environment, Tibet University, Lhasa 850032, Xizang, China University of Zimbabwe Harare Zimbabwe; 5 East African Herbarium, National Museums of Kenya, Nairobi P.O. Box 45166-00100, Kenya Tibet University Lhasa China; 6 Department of Botany, Jomo Kenyatta University of Agriculture and Technology, Nairobi P.O. Box 62000-00200, Kenya University of Chinese Academy of Sciences Beijing China; 7 Faculty of Agriculture, University of Zimbabwe, Harare, P.O. Box MP167, Zimbabwe Sino-Africa Joint Research Centre (SAJOREC), Chinese Academy of Sciences Wuhan China

**Keywords:** Africa, Flora of Kenya, Malpighiales, molecular phylogenetics, taxonomy

## Abstract

A new species, *Adenia sajoreciae* (Passifloraceae), from Kenya is described and illustrated. It is morphologically related to *A. ellenbeckii* but differs by being glabrous on all parts, except on the perianth lobes of male and female flowers (vs. subglabrous to pubescent throughout), bearing entire spinose-dentate leaves without laminar glands (vs. entire to deeply palmately 3–7-lobed or pinnatifid leaves with laminar glands), narrower flowers (vs. broader flowers), and globose, smaller fruits (vs. subglobose to ellipsoid, larger fruits). The phylogenetic analysis of *Adenia* based on ITS sequences placed three accessions of *A. sajoreciae* in a strongly supported and well-resolved clade (bootstrap support (BS) = 100%; posterior probability (PP) = 1.00), which is sister to *A. ellenbeckii* (BS = 99%; PP = 1.00), within *Adenia* sect. *Blepharanthes*. Based on both morphological and molecular evidence, *Adenia sajoreciae* N.Wei, S.W.Wang, Kirika & Q.F.Wang is described as a new species. Additionally, an updated identification key for species in the genus *Adenia* in Kenya is provided.

## Introduction

*Adenia* Forssk. (1775: 77) is the second-largest genus in Passifloraceae*s.s*. (De Wilde 1971; Melo and Guerra 2021), with 105 accepted species (Hearn 2006; POWO 2026), distributed throughout the Old World tropics and subtropics, including Africa, Madagascar, the Arabian Peninsula, India, and Southeast Asia (Melo and Guerra 2021; Mouafon and Katerere 2025). In Africa, *Adenia* is distributed across a wide ecological spectrum, ranging from arid savannas to humid forests (Hearn 2006; Mouafon and Katerere 2025). The Flora of Tropical East Africa (FTEA) recognizes 30 species of *Adenia* (De Wilde 1975; Hearn 2006), most of which belong to sect. *Blepharanthes*, a section characterized by thin, upturned glands (Hearn 2006). Of the 30 accepted species, approximately half occur in Kenya, with their distribution ranging from coastal lowlands to the highlands (De Wilde 1975; Beentje 1994, 2010; Agnew 2013). However, the recent discovery of *A. angulosa* G.W.Hu & Q.F.Wang by Ngumbau et al. (2017: 138) from the coastal forests of Kenya and Tanzania highlighted the potential for new taxa within the genus.

*Adenia* exhibits a wide range of growth forms: annual or perennial herbs, scandent shrubs, lianas, and pachycaul succulents, with the majority adapted to seasonally dry environments (De Wilde 1971; Hearn 2006; De Wilde and Eggli 2023). Its flowers are typically axillary cymes with cup-shaped hypanthia, free or basally united sepals, alternating and often reduced petals, and with or without coronas, which are sometimes vestigial (De Wilde 1974). *Adenia* species are predominantly dioecious and share a suite of unifying but variable traits, including axillary tendrils, spinose stems, and conspicuous petiolar glands (Hearn 2006; Melo and Guerra 2021). Hearn (2006) refined the infrageneric classification of *Adenia* by De Wilde (1971), recognizing six sections based on floral morphology, leaf form, extrafloral nectary position, and growth habits. A recent molecular phylogenetic analysis of over 60 *Adenia* species confirmed the monophyly of the genus within Passifloraceae*s.s*. but revealed complex interspecific relationships, particularly among the African lineages (Hearn 2006; Pignal et al. 2013). These findings highlighted the need for integrative taxonomic approaches that combine morphological and molecular data to resolve species delimitation within *Adenia*.

Based on the Flora of Kenya project (Zhou et al. 2017; Wei et al. 2026), botanical explorations in the Lake Baringo region were performed from 2023 to 2025. A peculiar individual of *Adenia* was encountered, which was first thought to be *A. ellenbeckii* Harms in  Engler and Drude (1921: 606). However, due to its entirely glabrous habit and entire, spinose-dentate leaves without laminar glands, it was clearly distinguishable from *A. ellenbeckii*. Upon further specimen consultation, as well as examination based on the local flora (FTEA) and local monographs (De Wilde 1975; Beentje 1994; Agnew 2013), this new specimen could not be assigned to any known accepted species in *Adenia*. Specimen examination through direct observation of morphological features at the East Africa Herbarium (EA) revealed that it was first collected in Baringo County, Kenya, in February 1990 and later documented in Samburu County, Kenya, but was never identified to the species level. Subsequently, a detailed morphological evaluation of the field specimens and photographs, along with molecular phylogenetic analyses of *Adenia* using ITS, supported its recognition as a new species.

## Materials and methods

### Molecular sampling and phylogenetics

Leaf tissues for molecular analysis were collected in the field and dried in silica gel (Chase and Hills 1991; Ruiz-Vargas et al. 2024). Following previous molecular phylogenetic analyses of *Adenia* (Hearn 2006; Pignal et al. 2013), the nuclear ribosomal internal transcribed spacer (ITS) was used for phylogenetic reconstruction. Three new ITS sequences from the new species were generated and integrated with published *Adenia* sequences downloaded from GenBank, for a total of 82 DNA sequences (Table 1). To assess the sectional placement of the newly described species within *Adenia*, ITS sequences of representative species from the major sections of the genus were analyzed. The ingroup comprised species belonging to the section to which the new taxon was hypothesized to belong, together with closely related sections. Species of *Adenia* sect. *Ophiocaulon* (Clade I *sensu*Hearn 2006) were used as an internal outgroup to root the phylogeny, as this section has consistently been recovered as an early-diverging, well-supported lineage in previous phylogenetic analyses of *Adenia* (Hearn 2006; Pignal et al. 2013).

**Table 1.** Taxa sampled with their corresponding voucher specimen information, GenBank accession numbers, and bibliographic references.

Species	Voucher specimen	Accession no.	References
*A. aculeata*	*Hearn cult1* (ARIZ)	DQ521302	Hearn 2006
*A. acuta*	*Hearn Mad007* (ARIZ)	DQ521328	Hearn 2006
*A. aff. antongilliana* DJH-2006 1	*Hearn Mad039* (ARIZ)	DQ521314	Hearn 2006
*A. aff. antongilliana* DJH-2006 2	*Hearn Mad020* (ARIZ)	DQ521313	Hearn 2006
*A. aff. antongilliana* DJH-2006 3	*Hearn Mad030* (ARIZ)	DQ521312	Hearn 2006
*A. ballyi*	*Hearn cult3* (ARIZ)	DQ521316	Hearn 2006
*A. barthelatii*	*Barthelat 1081* (P)	GU592816	Pignal et al. 2013
*A. boivinii*	*Hearn Mad023* (ARIZ)	DQ521346	Hearn 2006
*A. cardiophylla*	*Hearn cult42* (ARIZ)	DQ521295	Hearn 2006
*A. cissampeloides*	*Hearn 211119* (ARIZ)	DQ521370	Hearn 2006
*A. cladosepala*	*Hearn Mad011* (ARIZ)	DQ521331	Hearn 2006
*A. cordifolia*	*Mood 597* (Pupukea, Oahu)	DQ521298	Hearn 2006
*A. cynanchifolia*	*Breteler 12525* (WAG)	KC207258	Peccoud et al. 2013
*A. densiflora*	*Hearn Mad040* (ARIZ)	DQ521333	Hearn 2006
*A. digitata* 1	*Hearn 1040* (ARIZ)	DQ521361	Hearn 2006
*A. digitata* 2	*Hearn 1041* (ARIZ)	DQ521360	Hearn 2006
*A. digitata* 3	*Hearn cult4* (ARIZ)	DQ521363	Hearn 2006
*A. elegans*	*Hearn Mad053* (ARIZ)	DQ521332	Hearn 2006
*A. ellenbeckii*	*Hearn cult5* (ARIZ)	DQ521304	Hearn 2006
*A. fasciculata*	*Hearn Mad002* (ARIZ)	DQ521329	Hearn 2006
A. firingalavensis var. firingalavensiss	*Hearn Mad013* (ARIZ)	DQ521315	Hearn 2006
A. firingalavensis var. firingalavensiss	*Hearn ex. Specks* 3601 (ARIZ)	DQ526451	Hearn 2006
A. firingalavensis var. stylosa 1	*Hearn Mad028* (ARIZ)	DQ521337	Hearn 2006
A. firingalavensis var. stylosa 2	*Hearn cult28* (ARIZ)	DQ521336	Hearn 2006
A. firingalavensis var. stylosa 3	*Hearn cult34* (ARIZ)	DQ521335	Hearn 2006
A. fruticosa subsp. fruticosa	*Hearn 1029* (ARIZ)	DQ521319	Hearn 2006
*A. glauca*	*Hearn 1035* (ARIZ)	DQ521348	Hearn 2006
A. globosa subsp. pseudoglobosa	Huntington BG 78550 (ARIZ)	DQ521317	Hearn 2006
*A. goetzei*	Hearn cult8 (ARIZ)	DQ521355	Hearn 2006
*A. gummifera*	*Hearn 1015* (ARIZ)	DQ521371	Hearn 2006
A. hastata var. glandulifera	*Hearn 1008* (ARIZ)	DQ521301	Hearn 2006
A. hastata var. hastata	*Hearn 1027* (ARIZ)	DQ521300	Hearn 2006
*A. heterophylla*	s.n.	DQ499119	Wright et al. 2006
A. heterophylla subsp. australis	*Hearn cult11* (ARIZ)	DQ521299	Hearn 2006
A. heterophylla var. heterophylla	*Hearn T3* (ARIZ)	DQ521297	Hearn 2006
*A. hondala*	*Hearn cult12* (ARIZ)	DQ521309	Hearn 2006
*A. inermis*	*Hearn cult13* (ARIZ)	DQ521303	Hearn 2006
*A. isaloensis*	*Hearn Mad046* (ARIZ)	DQ521325	Hearn 2006
*A. karibaensis*	*Hearn cult14* (ARIZ)	DQ521320	Hearn 2006
*A. keramanthus*	*Hearn 201116* (ARIZ)	DQ521308	Hearn 2006
*A. kirkii* 1	*Hearn 261131* (ARIZ)	DQ521359	Hearn 2006
*A. kirkii* 2	*Hearn 181113* (ARIZ)	DQ521358	Hearn 2006
*A. kirkii* 3	*Krosnick 257* (OS)	AY632698	Krosnick and Freudenstein 2005
A. lanceolata subsp. scheffleri	*Hearn 201115* (ARIZ)	DQ521367	Hearn 2006
*A. lapiazicola*	*Hearn Mad026* (ARIZ)	DQ521341	Hearn 2006
*A. letouzeyi*	*se 39-1490* (Meise)	DQ521350	Hearn 2006
*A. lindiensis*	*Hearn 181110* (ARIZ)	DQ521368	Hearn 2006
*A. lobata*	*De Wilde 873* (Meise)	DQ521351	Hearn 2006
*A. longestipitata*	*Hearn Mad042* (ARIZ)	DQ521327	Hearn 2006
*A. mannii*	*Escobar 92-38* (ARIZ)	DQ521353	Hearn 2006
*A. metriosiphon*	*Hearn cult18* (ARIZ)	DQ521369	Hearn 2006
*A. monadelpha*	*Hearn Mad050* (ARIZ)	DQ521344	Hearn 2006
A. olaboensis var. olaboensis	*Hearn Mad016* (ARIZ)	DQ521323	Hearn 2006
A. olaboensis var. parva	*Hearn Mad052* (ARIZ)	DQ521324	Hearn 2006
*A. ovata*	*Hearn cult19* (ARIZ)	DQ521356	Hearn 2006
*A. pachyphylla*	*Hearn cult20* (ARIZ)	DQ521330	Hearn 2006
*A. pechuelii*	*Hearn cult21* (ARIZ)	DQ521321	Hearn 2006
*A. peltata*	*Hearn cult22* (ARIZ)	DQ521347	Hearn 2006
A. penangiana var. parvifolia	*Hearn T6* (ARIZ)	DQ521296	Hearn 2006
*A. perrieri*	*Hearn Mad048* (ARIZ)	DQ521322	Hearn 2006
*A. pulchra*	Specks s.n.	DQ521366	Hearn 2006
*A. pyromorpha*	*Hearn Mad061* (ARIZ)	DQ521326	Hearn 2006
*A. racemosa*	*Hearn 261132* (ARIZ)	DQ521311	Hearn 2006
*A. refracta*	*Hearn Mad008* (ARIZ)	DQ521345	Hearn 2006
*A. repanda*	*Hearn 1023* (ARIZ)	DQ521354	Hearn 2006
*A. rumicifolia*	*Hearn 41241* (ARIZ)	DQ521352	Hearn 2006
*A. sajoreciae* 1	*Rachel BFFP-711* (EA, K)	PZ392830	This study
*A. sajoreciae* 2	*Luke et al. 14016* (EA, K)	PZ392831	This study
*A. sajoreciae* 3	*Rachel BFFP-681* (EA, K)	PZ392832	This study
*A. schweinfurthii*	*Hearn cult25* (ARIZ)	DQ521310	Hearn 2006
*A. sphaerocarpa* 1	*Hearn Mad014* (ARIZ)	DQ521339	Hearn 2006
*A. sphaerocarpa* 2	*Hearn cult26* (ARIZ)	DQ521338	Hearn 2006
*A. spinosa*	*Hearn 1022* (ARIZ)	DQ521349	Hearn 2006
*A. stenodactyla*	*Hearn 3515* (ARIZ)	DQ521365	Hearn 2006
*A. stricta*	*Hearn cult2* (ARIZ)	DQ521307	Hearn 2006
*A. subsessilifolia*	*Hearn Mad051* (ARIZ)	DQ521334	Hearn 2006
*A. trisecta*	*Hearn cult31* (ARIZ)	DQ521357	Hearn 2006
*A. venenata*	*Hearn cult29* (ARIZ)	DQ521318	Hearn 2006
*A. volkensii* 1	*Hearn cult30* (ARIZ)	DQ521305	Hearn 2006
A. wightiana subsp. africana	*Hearn 18119* (ARIZ)	DQ521373	Hearn 2006
A. wightiana subsp. wightiana	*Krosnick* (OSU)	DQ521374	Hearn 2006
*A. wilmsii*	*Hearn 1028* (ARIZ)	DQ521362	Hearn 2006

Total genomic DNA was extracted from ca. 10–30 mg of silica-dried leaf tissue using a modified cetyltrimethylammonium bromide (CTAB) method (Doyle and Doyle 1987; Quiñones et al. 2024). The primer design and PCR conditions followed Pignal et al. (2013). The PCR reactions were set up in 25 µL volumes, and amplification success was checked on agarose gels. Amplicons were purified and sequenced bidirectionally by Sanger sequencing (Crossley et al. 2020). Sequences were assembled and edited in Geneious Prime (Kearse et al. 2012). All sequences were aligned with MAFFT (Katoh et al. 2019) and trimmed using TrimAl (Capella-Gutiérrez et al. 2009) in PhyloSuite v1.2.3 (Zhang et al. 2020). Alignments were visually inspected and manually adjusted in Geneious (Winn et al. 2025). Phylogenetic inference was conducted using both maximum likelihood (ML) and Bayesian inference (BI). ModelFinder (Kalyaanamoorthy et al. 2017) was used to select the best-fit model based on the Bayesian information criterion (BIC) for ML and BI analyses. ML analyses were performed in IQ-TREE (Nguyen et al. 2015), with support assessed using 1,000 bootstrap replicates. BI analyses were conducted in MrBayes (Ronquist et al. 2012), with two independent runs of four chains each, run until convergence (average standard deviation of split frequencies < 0.01; effective sample size (ESS) > 200). Trees were visualized and annotated in iTOL (Letunic and Bork 2024). The phylogenetic tree reconstruction adopted the detailed description by Wei et al. (2020). All newly generated sequences were deposited in GenBank.

Exact nucleotide differences, substitutions, and indels in the ITS region between the putative new species and *A. ellenbeckii* were quantified using MEGA12 (Kumar et al. 2024). ITS sequences of the new species and *A. ellenbeckii* were aligned in MEGA12 (Kumar et al. 2024) using MUSCLE (Edgar 2004) with default parameters. The alignment was manually inspected, and terminal regions with missing data were trimmed to retain a shared overlapping region of 495 bp. Variable nucleotide positions were identified by direct inspection of the alignment in MEGA’s Alignment Explorer. Fixed substitutions and indels distinguishing the taxa were recorded manually. Pairwise genetic distances were calculated using *p*-distance with pairwise deletion of gaps.

### Taxonomy

Morphological observations and measurements were made on living plants in the field, and photographs were taken using a Nikon D810 digital DSLR camera. Additional floral and vegetative structures were examined and photographed using a Zoom Stereo Microscope TS-10W (PDV, China) (Qiu et al. 2025). Herbarium specimens were collected and deposited at the East African Herbarium (EA) and Wuhan Institute of Botany Herbarium (HIB), while fresh flowers for floral dissection were picked and preserved in 70% ethanol (Lv et al. 2024). Comparative morphological work included the examination of digitized specimens at the Royal Botanic Gardens, Kew (K), the Natural History Museum (BM), the National Botanic Garden of Belgium (BR), the Muséum National d’Histoire Naturelle (P), and JSTOR Global Plants (https://www.jstor.org/). On-site specimen examination was conducted at EA and HIB. Morphological characters were measured with digital calipers to the nearest 0.1 mm. Dissections of the floral parts were made under a Zoom Stereo Microscope TS-10W stereomicroscope (PDV, China), as described by Qiu et al. (2025). Botanical terminology is presented according to De Wilde (1975), Hearn (2006), and Beentje (2010). Descriptions and character states are based on the range of variation observed in all 16 herbarium specimens and living materials examined. Morphological descriptions and diagnostic comparisons follow the standard guidelines of the International Code of Nomenclature (Turland et al. 2025). The conservation assessment followed the IUCN Red List Guidelines Version 16 (IUCN Standards and Petitions Committee 2024). AOO and EOO were calculated in GeoCAT (Bachman et al. 2011) using a 2 × 2 km grid cell method, with threat-defined locations identified based on the most serious plausible threats.

## Results

### Molecular phylogenetics

**Figure 1.**
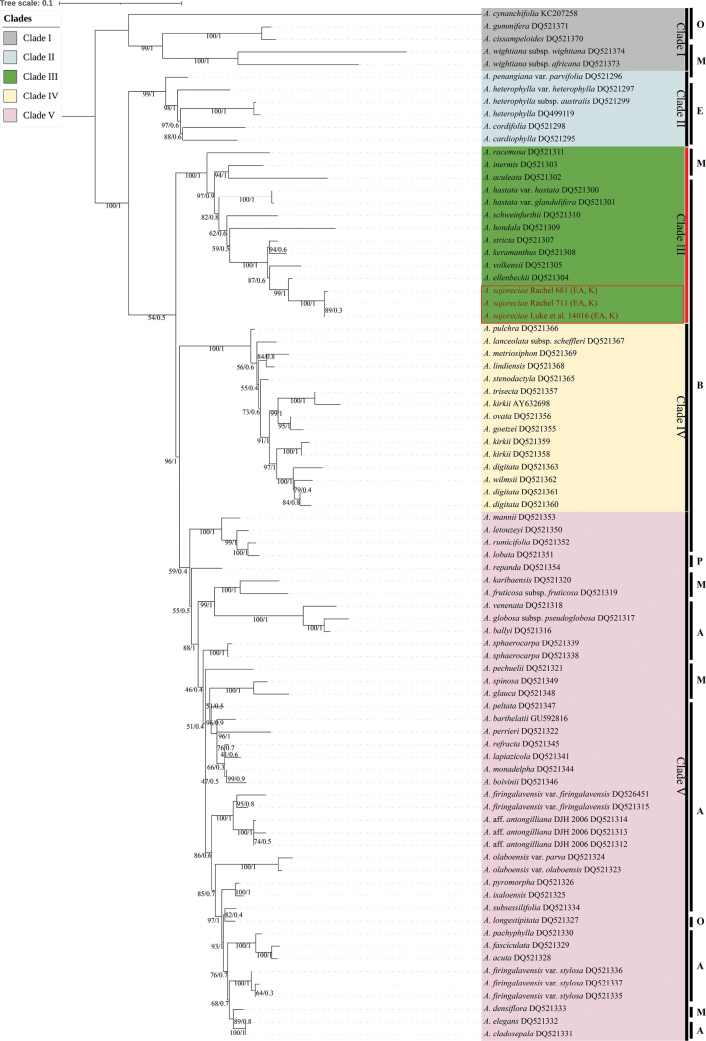
Maximum likelihood phylogenetic tree of *Adenia* based on ITS sequences from 82 accessions. Bootstrap support (BS) and posterior probability (PP) values are indicated along the branches; accessions of *A. sajoreciae* are indicated in red font. Section key: **A** = *Adenia*; **B** = *Blepharanthes*; **M** = *Microblepharis*; **O** = *Ophiocaulon*; **P** = *Paschanthus*.

As shown in Fig. 1, the ML phylogenetic tree of *Adenia* based on ITS sequences from 82 accessions provides an evolutionary framework for the new species, with both bootstrap support (BS) and posterior probability (PP) values indicated along the branches. The phylogeny recovers five principal clades (Clades I–V), reflecting broad structuring within the genus and grouping taxa according to the sampled sectional circumscriptions (*Adenia*, *Blepharanthes*, *Microblepharis*, *Ophiocaulon*, and *Paschanthus*). The deeper nodes separate several major lineages with generally moderate to strong support, although support among some internal relationships is variable. Sectional circumscriptions are only partly congruent with the recovered topology, as some sections are distributed across multiple clades rather than forming exclusive monophyletic groups, whereas others are recovered as more cohesive lineages. Within the phylogeny, Clade V and sections *Adenia* and *Blepharanthes* represent the most species-rich assemblages. Within this framework, the three accessions of *A. sajoreciae* (highlighted in red font in Fig. 1) form a monophyletic clade, deeply nested within sect. *Blepharanthes* (Clade III, marked with a red line) (BS = 100%, PP = 1.00). This clade is recovered as sister to *A. ellenbeckii*, with strong statistical support (BS = 99%, PP = 1.00). The ITS alignment of three accessions of *A. sajoreciae* and one accession of *A. ellenbeckii*, trimmed to the overlapping region shared by all accessions, comprised 495 bp (Suppl. material 1). Comparison between *A. sajoreciae* (three accessions) and *A. ellenbeckii* revealed consistent nucleotide differences despite strong phylogenetic clustering. These include two fixed nucleotide substitutions (C→G) at alignment positions 14 and 57 and a fixed indel at position 462. One accession of *A. sajoreciae* exhibited an additional substitution at position 146, interpreted as intraspecific polymorphism. Pairwise *p*-distances between *A. sajoreciae* and *A. ellenbeckii* ranged from 0.0020 to 0.0061, corresponding to approximately 1–3 nucleotide differences across the 495 bp alignment and providing corroborative molecular evidence.

### Taxonomy

#### Adenia sajoreciae

Taxon classificationPlantaeMalpighialesPassifloraceae

N.Wei, S.W.Wang, Kirika & Q.F.Wang sp. nov.

0ADE0A6A-2B50-5756-A706-E9B80EF9D41A

urn:lsid:ipni.org:names:77381436-1

Figs 2, 3, 4

##### Type.

Kenya. • Baringo County, Kapkoror, the headquarters of Baringo Fuel & Fodder Project (BFFP), 28 Feb. 1990, *Rachel BFFP-682* (holotype: EA!; isotype: K barcode K005188453!).

**Figure 2.**
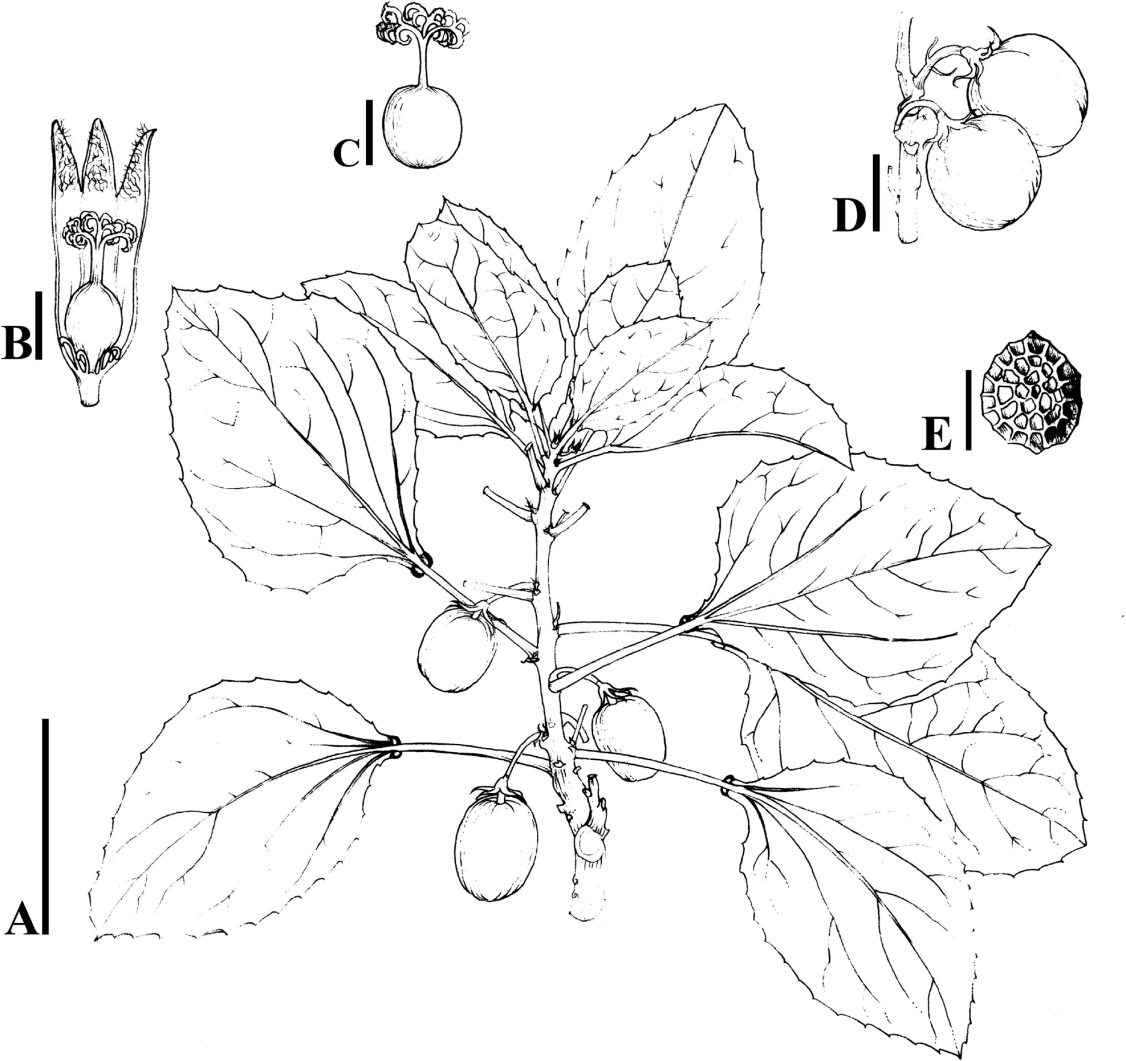
*Adenia sajoreciae*. **A**. Leafy fruiting shoot; **B**. Female flower; **C**. Ovary, style, and stigma; **D**. Fruits; **E**. Pitted seed. Scale bars: 5 cm (**A**); 5 mm (**B, C, E**); 2 cm (**D**). Drawing by Nan Jia.

**Figure 3.**
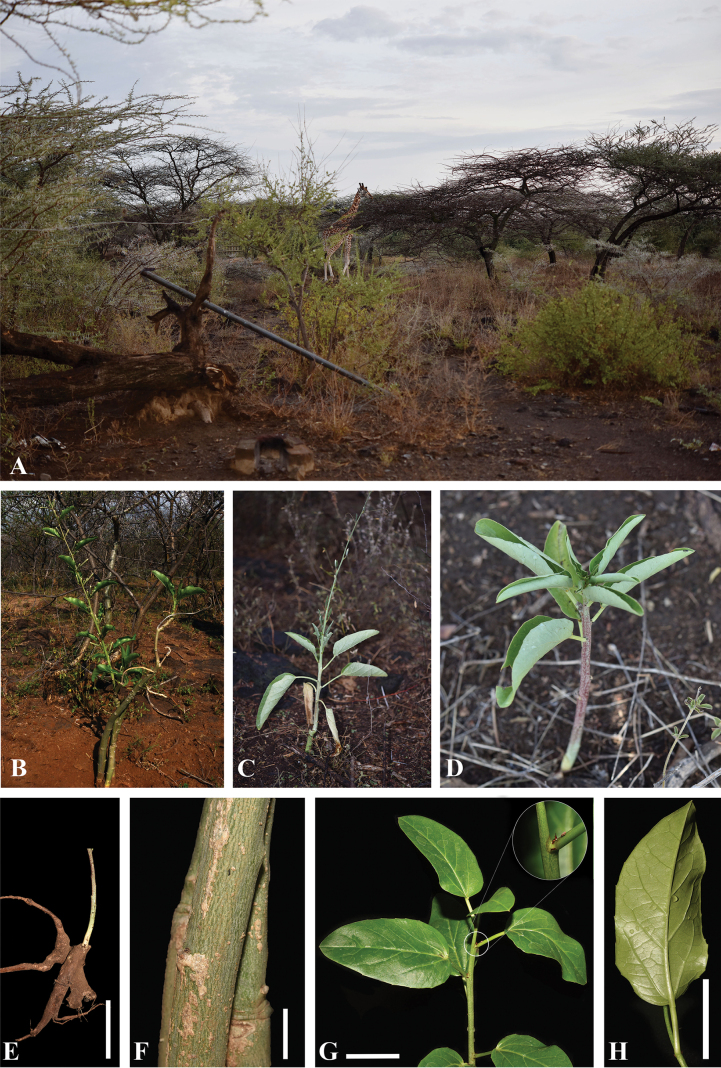
*Adenia sajoreciae*. **A**. Habitat within dry *Acacia*–*Commiphora* woodland; **B–D**. Growth habits; **E**. Root; **F**. Succulent stems; **G**. Leafy shoot showing the position of the stipule; **H**. Abaxial surface of spinose-dentate leaf. Scale bars: 5 cm (**E**); 2 cm (**F–H**).

**Figure 4.**
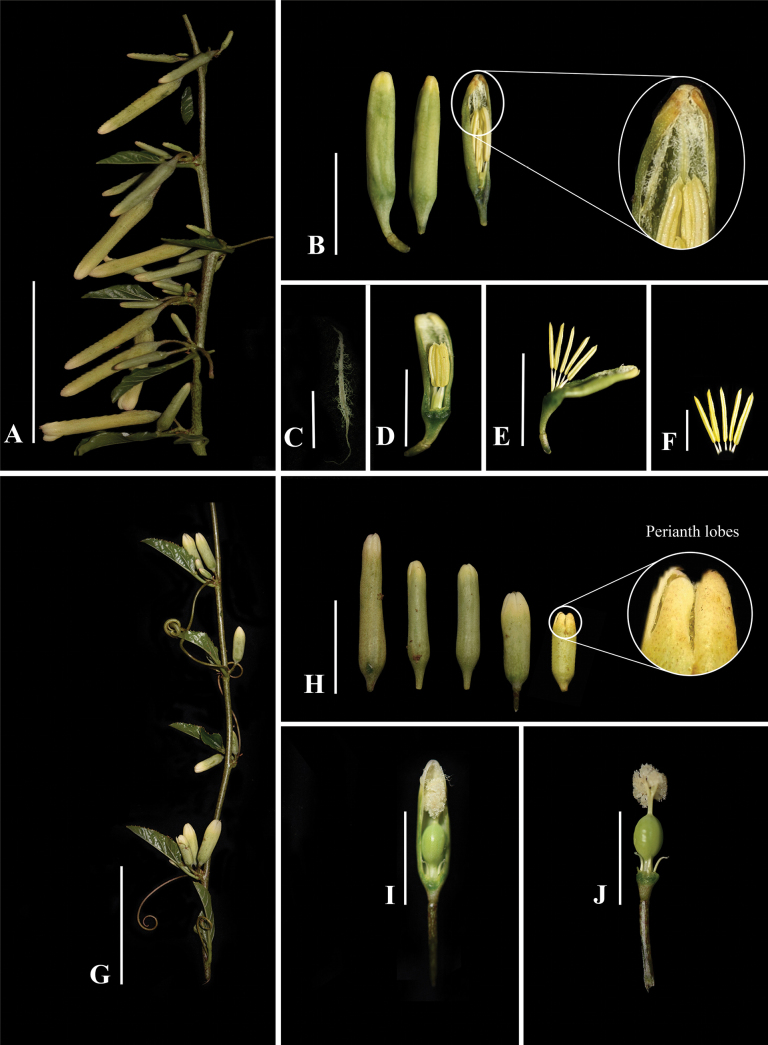
*Adenia sajoreciae*. **A**. Male inflorescence; **B**. Male flowers; **C**. Pistillode; **D, E**. Dissected male flower, showing androecium with five anthers; **F**. Anthers, detached; **G**. Female inflorescence; **H**. Female flowers; **I**. Dissected female flower, showing receptacle, perianth, ovary, and style; **J**. Female flower showing receptacle, ovary, style, and stigma. Scale bars: 5 cm (**A, G**); 1 cm (**B, D, E, I, J**); 2 cm (**H**); 5 mm (**C, F**).

##### Diagnosis.

*Adenia sajoreciae* is close to *A. ellenbeckii* but can be distinguished by its entirely glabrous habit (excluding the perianth lobes) (vs. subglabrous to pubescent), caducous subulate stipules (vs. persistent triangular stipules), entire leaves (vs. entire to deeply palmately 3–7 lobed or pinnatifid), absence of laminar glands on leaf blade (vs. presence of lamina glands on leaf blade), spinose-dentate leaf margins (vs. dentate or dissected leaf margin), male flowers 2–3 mm in width (vs. 3.0–6.5 mm in width), female flowers 3–5 mm in width (vs. 4.5–8.0 mm in width), globose fruits, 2–3 cm in diameter when mature (vs. subglobose to ellipsoid, 3.5–5.5 × 3.0–4.5 cm).

##### Description.

Perennial, entirely glabrous herb or scandent subshrub, to 1.7–2.0 m high, arising from a tuberous rootstock. ***Stems*** succulent at base, to ca. 0.3 m high; older stems striate, corky, cylindrical; young shoots slender, terete, green, sparsely lenticellate. ***Tendrils*** filiform, to 10 cm long, green with brownish cast, opposite or adjacent to the inflorescences. ***Stipules*** minute, subulate, caducous. ***Petiole*** 1–9 cm, with 2 sessile, opposite petiolar blade-junction glands. ***Leaves*** simple, entire, narrowly-broadly ovate, 6–12 × 2–9 cm, laminar glands absent, margins spinose-dentate; base obtuse to subcordate, apex acute to shortly acuminate, adaxial surface dark green, abaxial paler; ***venation*** cladodromous with brochidodromous secondaries. ***Inflorescence*** an axillary lax raceme, in both male and female flowers. ***Peduncle not sessile***, 1.5–3.2 cm long in male flowers, and to 3.0–5.5 cm long in female flowers. ***Male flowers*** 1–7 together, tubular-cylindrical, 16–22 × 2–3 mm; ***hypanthium*** cylindrical to narrowly cup-shaped, 3.0–4.5 mm; ***perianth lobes*** five, pubescent, triangular, 2–3 mm long; ***perianth tube*** white fimbriate from the inner upper ¼, extending to cover the inner part of the lobes; ***petals*** five, linear-lanceolate, 8–12 mm long, greenish-yellow; ***stamens*** five, ***filaments*** five, whitish, ca. 3 mm; ***anthers*** five, bright yellow, linear, versatile, 5–6 mm long; pistiloids, ca. 1.5 cm; receptacle cupular, ca. 1 cm. ***Female flowers*** 1–4 together, tubular-cylindrical, 12–36 × 3–5 mm; ***hypanthium*** cylindrical to narrowly cup-shaped, 3–4 mm; ***perianth lobes*** five, pubescent, triangular, 1.5–3.0 mm long; ***perianth tube*** as in male flowers; ***petals*** five, linear-lanceolate, 8–12 mm long, greenish-yellow; ***gynophore*** 1–2 mm; ***ovary*** ellipsoid-prolate, 4–5 × 2–3 mm; ***styles*** 3, 7–9 mm long, fused at base, lobed–papillate, 3.5–4.0 mm in diameter; ***staminodes*** slender, ca. 3 mm long; receptacle cupular, ca. 1 cm. ***Fruit*** a berry, globose, 2–3 cm in diameter when mature, brown when ripe, spongy inside. ***Seeds*** ovate, ca. 7 mm long, testa pitted.

##### Specimens examined

**(paratypes)**. Kenya. • Baringo County, Kapkoror, the headquarters of BFFP, *Rachel BFFP-681* (EA!, K barcode K005188451!, PZ392832); • Baringo County, Kapkoror, the headquarters of BFFP, 19 Mar. 1990, *Rachel BFFP-711* (EA!, K barcode K005188452!, PZ392830); • Baringo County, Ruko Community Conservancy, 0°38'32.73"N, 36°7'38.01"E, Alt. 971 m, 10 Mar. 2025, *Kipkemoi E. KE-KE-002* (HIB!); • topotyp., 23 Jan. 2024, *Wang et al. KE-WSW83* (HIB!); • Samburu County, Lonjorin, along Tuum Lonjorin road, 2°17'45.2"N, 36°47'47.0"E, Alt. 1220 m, 6 Feb. 2010, *Luke Q. et al. 14016* (EA!, K barcode K005188459!, PZ392831).

##### Other specimens examined for *Adenia ellenbeckii*.

**Kenya**. • Dandu, 3°26'N, 39°54'E, Alt. 750 m, 8 Apr. 1952, *Gilbert 12722* (EA!, K barcode K005188223!); • Dandu, 3°26'N, 39°54'E, Alt. 750 m, 14 Apr. 1952, *Gilbert 12787* (EA!, K barcode K005188219!); • Furroli, 3°42'N, 38°00'E, Alt. 950 m, 17 Sept. 1952, *Gillette 13926* (EA!, K barcode K005188216!, K005188217!); • Isiolo, 0°23'N, 37°32'E, Alt. 1080 m, 19 Feb. 1953, *Gilbert 15149* (K barcode K005188221!); • Huri Hills, 3°24'N, 37°45'E, 25 Feb. 1963, *Bally 12516* (EA!, K barcode K005188225!); • Machakos District, 12 Nov. 1968, *Greenway & Duvigneaud 12622* (EA!, K barcode K005188218!); • Nairobi, 18 Jan. 1943, *Bally 2201* (EA!, K!); • Marsabit–Isiolo Road, 1°37'N, 37°49'E, Alt. 500 m, 1 Nov. 1978, *Gilbert et al. 5290* (EA!, K barcode K005188213!); • Marsabit, outskirts of Serolewi, Marsabit–Isiolo Road, Alt. ca. 1613 m, 2 Nov. 1978, *Gilbert et al. 5295* (EA!, K barcode K005188214!); • Marsabit, outskirts of Serolewi, Marsabit–Isiolo Road, 1°07'N, 37°37'E, Alt. 500 m, 2 Nov. 1978, *Gilbert et al. 5294* (EA!, K barcode K005188215!); • Mt. Kulal, Alt. 1558–1798 m., 8 Oct. 1947, *Bally 5488* (EA!, K barcode K005188220!).

##### Etymology.

The specific epithet “*sajoreciae*” is in recognition of the Sino-Africa Joint Research Center (SAJOREC), which was established in Kenya and headquartered in Nairobi in 2013 by the Chinese Academy of Sciences, for its significant contribution to plant diversity documentation and conservation in Africa.

##### Phenology.

The new species was observed flowering in the field between January and March, while fruiting has been observed between February and March.

##### Distribution and habitat.

**Figure 5.**
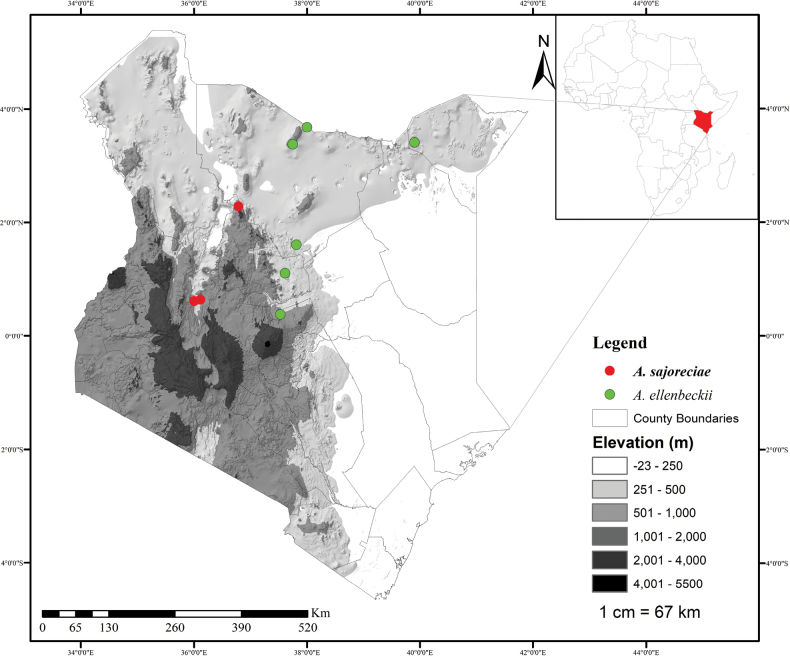
Distribution map of *Adenia sajoreciae* (red dots) and *A. ellenbeckii* (green dots) in Kenya.

*Adenia sajoreciae* has so far been recorded from Kapkoror Sub-County, Baringo Fuel & Fodder Project (BFFP) headquarters, Ruko Community Conservancy (a protected area managed by the local community) in Baringo County, Kenya, and Lonjorin in Samburu County, Kenya (Fig. 5). The species was documented from well-drained, rocky soils in open, dry bushlands with minimal anthropogenic disturbance, characterized by *Vachellia* spp., *Boscia* spp., *Grewia* spp., *Commiphora* spp., and *Adenium obesum* (Forssk.) Roem. & Schult., in association with xerophytic shrubs and succulent species, at an elevation of ca. 900–1,200 m.

##### Conservation status.

*Adenia sajoreciae* is assessed as Endangered (EN; B2ab(ii, iii)) following the IUCN Red List Categories and Criteria. The AOO was estimated at 12 km^2^ and EOO at 943.1 km^2^ using a 2 × 2 km grid cell method in GeoCAT (Bachman et al. 2011), satisfying the EN threshold of AOO < 500 km^2^. The assessment is justified by (a) ≤ 5 threat-defined locations representing severe fragmentation and (b) a projected continuing decline in (ii) AOO and (iii) habitat area, extent, and quality. The species is known from five herbarium specimens and one population of approximately 15 individuals restricted to well-drained, rocky soils in open, dry bushlands. Ongoing habitat degradation from livestock grazing and possible airstrip expansion indicates a continuing decline in its suitable habitat.

##### Uses.

Its leaves are cooked as vegetables in Baringo County, Kenya.

##### Notes.

**Figure 6.**
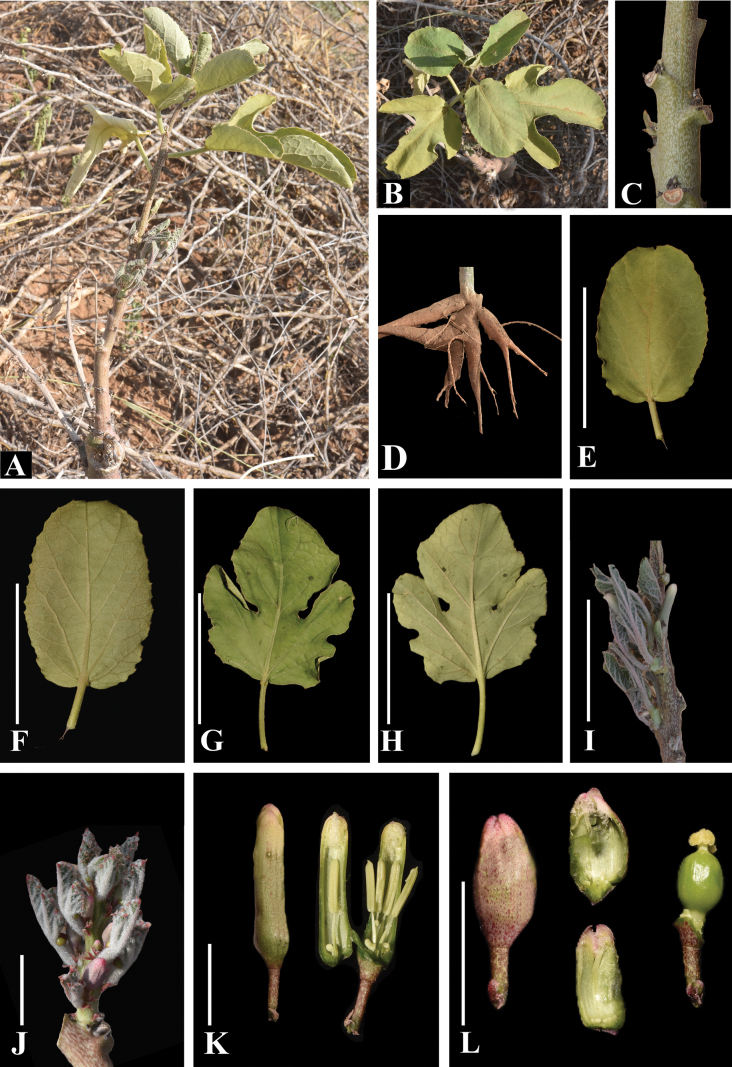
*Adenia ellenbeckii* Harms. **A, B**. Habit; **C**. Stem; **D**. Tuberous root; **E, F**. Adaxial and abaxial leaf surfaces (entire leaf); **G, H**. Adaxial and abaxial leaf surfaces (lobed leaf); **I**. Male inflorescence with pubescent young leaves; **J**. Female inflorescence with pubescent young leaves; **K**. Male flowers; **L**. Female flowers. Scale bars: 5 cm (**E–I**); 2 cm (**J**); 1 cm (**K, L**).

The new species, *A. sajoreciae* (Figs 2, 3, 4), is morphologically close to *A. ellenbeckii* (Fig. 6) but differs mainly in leaf morphology. *A. sajoreciae* bears entire leaves without laminar glands and with spinose-dentate margins (vs. deeply palmately 3–7-lobed or pinnatifid leaves bearing submarginal laminar glands and non-spinose margins), glabrous indumentum except for the perianth lobes (vs. subglabrous to pubescent throughout), minute, subulate, caducous stipules (vs. persistent, triangular, dark brown stipules), male flowers 2–3 mm in width and female flowers 3–5 mm in width (vs. male flowers 3.0–6.5 mm in width and female flowers 4.5–8.0 mm in width), and smaller, globose fruit, 2–3 cm (vs. larger, subglobose-ellipsoid fruit, 3.5–5.5 × 3.0–4.5 cm). The diagnostic characters separating the two species are summarized in Table 2. A morphological key to the identification of *Adenia* in Kenya, including the new species, is presented below.

**Table 2.** Morphological comparison between *A. sajoreciae* and *A. ellenbeckii.*

Character	*A. sajoreciae*	*A. ellenbeckii*
**Habit**	Herb or scandent subshrub, 170–200 cm tall	Shrub or herb up to 150 cm tall
**Indumentum**	Vegetative parts glabrous; perianth lobes pubescent	Subglabrous to pubescent throughout
**Leaf outline**	Entire	Entire to deeply palmately 3–7-lobed or pinnatifid
**Leaf margin**	Spinose-dentate	Dentate or dissected, never spinose
**Leaf surface**	Adaxial dark green, glabrous; abaxial distinctly paler, glabrous	Green, ± pubescent on both surfaces
**Leaf venation**	Cladodromous, secondaries brochidodromous, main veins ca. 5 cm	3–5 main nerves from near the base or ± pinninerved
**Laminar glands**	Absent	Submarginal, 0–6
**Leaf size**	6–12 × 2–9 cm	2–17 × 2–12 cm
**Petiole**	1–9 cm long, glabrous	1–7 cm long, pubescent
**Stipule**	Subulate, brown, minute, caducous	Triangular, dark brown, persistent
**Inflorescence**	Male 1–7-flowered, female 1–4-flowered	Male 1–10-flowered, female 1–3-flowered
**Flower width**	2–3 mm in male, 3–5 mm in female	3.0–6.5 mm in male, 4.5–8.0 mm in female
**Fruit**	Globose, 2–3 cm	Subglobose to ellipsoid, 3.5–5.5 × 3.0–4.5 cm

### Key to *Adenia* species in Kenya (modified from De Wilde 1975)

**Table:** 

1	Shrubs with massive green stem-base	**2**
–	Herbs or shrubs without massive stem-base	**3**
2	Branches spinescent	***A. globosa***
–	Plants unarmed	***A. venenata***
3	Leaf blade with a basal appendage bearing gland(s); corona, if present, never fimbriate or hairy; flowers without disc glands	**4**
–	Leaf blade without basal appendage; corona, if present, fimbriate or hairy; flowers with disc glands	**8**
4	Leaf glands, when present, confined to vein axils	***A. bequaertii***
–	Leaf glands not confined to vein axils	**5**
5	Leaves entire; venation acrodromous, secondary veins arching to apex	***A. stolzii***
–	Leaves often lobed; venation more or less straight, upper secondary veins ending in marginal glands	**6**
6	Leaves never lobed; venation obscure on adaxial surface	***A. cissampeloides***
–	Leaves often lobed; venation clearly visible on adaxial surface	**7**
7	Older stems terete; juvenile leaves shallowly 3-lobed; fruit broadly ovoid to elliptic, cross-section round	***A. gummifera***
–	Older stems 3–5-angled; juvenile leaves deeply (3–)5(–7)-lobed; fruit narrowly ovoid, 6-angled	***A. angulosa***
8	Flowers > 8 mm long; perianth with fused tube	**9**
–	Flowers < 5 mm long; sepals free	***A. wightiana***
9	Perianth lobes and sepals of ♂ flowers glabrous or only minutely denticulate; leaves usually glabrous	**10**
–	Perianth lobes or sepals of ♂ flowers pubescent to long-villous; leaves glabrous or pubescent, entire or lobed	**13**
10	Hypanthium < 5 mm wide, narrower than perianth tube	**11**
–	Hypanthium 5–15 mm wide, equalling perianth tube	**12**
11	Sepal tube and petals of ♂ flowers > 10 mm; leaf base emarginate or truncate	***A. metriosiphon***
–	Sepal tube and petals of ♂ flowers < 10 mm; leaf base rounded or cuneate	***A. lanceolata***
12	Anthers > 5 mm long, exceeding filaments; fruit pyriform	***A. rumicifolia***
–	Anthers ≤ 5 mm, shorter than filaments; fruit globose to ellipsoid	***A. schweinfurthii***
13	Stipules subulate, caducous; laminar glands absent; leaves entire, margin spinose-dentate; plants entirely glabrous (excluding perianth lobes); male flowers 2–3 mm wide; female flowers 3–5 mm wide; fruit globose, 2.5–3.0 × 2.0–2.5 cm; seeds ovate	***A. sajoreciae***
–	Stipules triangular, persistent; laminar glands present or absent; leaves entire to lobed, margin dentate or dissected; plants ± pubescent to glabrous	**14**
14	Plants climbing; leaves 3–7-lobed; basal glands stipitate; anthers 8–12 mm long	***A. volkensii***
–	Plants climbing or ± pubescent; leaves entire to lobed; stipules triangular, persistent; male flowers various; fruit various	**15**
15	Plants ± pubescent; leaves entire to deeply palmately 3–7-lobed or pinnatifid; leaf margins dentate or dissected; laminar glands present; male flowers 3.0–6.5 mm wide; female flowers 4.5–8.0 mm wide; fruit subglobose to ellipsoid, 3.5–5.5 × 3.0–4.5 cm; seeds ovate	***A. ellenbeckii***
–	Plants glabrous to sparsely pubescent; leaves entire or 2–5-lobed, base peltate or auriculate; margin not spinose-dentate; laminar glands absent; seeds ovate	***A. lindiensis***

## Discussion

The molecular phylogenetic reconstruction based on ITS sequences placed *A. sajoreciae* within *Adenia* sect. *Blepharanthes* (Clade III), a section characterized by relatively long tubular to urceolate flowers and common upturned glands at the base of mature leaf blades and at the petiolar apices (Hearn 2006). The overall topology recovered was congruent with previous systematic treatments that grouped the East African tuberous, herbaceous-to-subwoody climbers of *Adenia* as an assemblage (Hearn 2006; Pignal et al. 2013). Morphologically, *A. sajoreciae* is defined by a distinctive combination of vegetative and floral characters that distinguish it from other members of *Adenia* in East Africa. While some traits may be shared with some congeners, it exhibits clear morphological discontinuities from its closest relative, *A. ellenbeckii* (De Wilde 1971; Hearn 2006). Phylogenetically, the fixed ITS divergence between *A. sajoreciae* and *A. ellenbeckii* is typical of closely related species within *Adenia* and, together with morphological evidence, supports the recognition of the new taxon.

## Conclusion

The distinct morphological characteristics of *Adenia sajoreciae*, together with molecular divergence based on ITS data, support its recognition as a new species. Its provisional conservation assessment as Endangered (EN) underscores the urgency of continued floristic exploration and targeted conservation efforts within its range. Further floristic surveys are particularly needed in northern Kenya, a region that remains comparatively underexplored despite its potential significance for biodiversity discovery and conservation.

## Supplementary Material

XML Treatment for Adenia sajoreciae

## Additional information

### Artificial Intelligence (AI) use

The authors accept full responsibility for the content of the manuscript, including the disclosure of any use of AI.

No AI tools were used in the preparation of this manuscript.

### Author ORCIDs

E. Kipkemoi https://orcid.org/0009-0006-7766-5141

F. Munyao Mutie https://orcid.org/0000-0001-7666-2922

X.-D. Qiu https://orcid.org/0009-0007-2464-6921

P.M. Kirika https://orcid.org/0000-0002-4318-9339

J.K. Muchuku https://orcid.org/0000-0001-9095-4681

E. Nyakudya https://orcid.org/0000-0002-2846-4427

X. Yan https://orcid.org/0000-0003-4460-8117

S.-W. Wang https://orcid.org/0000-0002-8785-5065

N. Wei https://orcid.org/0000-0002-0313-3912

Q.-F. Wang https://orcid.org/0000-0001-9143-8849

### Data availability

All of the data that support the findings of this study are available in the main text or Supplementary Information.

## Supplementary materials

Supplementary material 1 ITS trimmed alignment dataset of *Adenia sajoreciae* and *A. ellenbeckii* Data type fas https://binary.pensoft.net/file/1678683This dataset is made available under the Open Database License (http://opendatacommons.org/licenses/odbl/1.0/). The Open Database License (ODbL) is a license agreement intended to allow users to freely share, modify, and use this Dataset while maintaining this same freedom for others, provided that the original source and author(s) are credited.Emmanuel Kipkemoi, Fredrick Munyao Mutie, Xiang-Dong Qiu, Paul Muigai Kirika, John Kamau Muchuku, Elijah Nyakudya, Xue Yan, Sheng-Wei Wang, Neng Wei, Qing-Feng Wang
